# Association between neurological soft signs, temperament and character in patients with schizophrenia and non-psychotic relatives

**DOI:** 10.7717/peerj.1651

**Published:** 2016-04-26

**Authors:** Liliana Galindo, Francisco Pastoriza, Daniel Bergé, Anna Mané, Marisol Picado, Antonio Bulbena, Patricia Robledo, Victor Pérez, Oscar Vilarroya, Claude Robert Cloninger

**Affiliations:** 1 Neuropsychiatry and Addiction Institute, Parc de Salut Mar, Barcelona, Spain; 2 Neurosciences Research Programme, Hospital del Mar Medical Research Institute (IMIM), Barcelona, Spain; 3 Departament de Psiquiatria i Medicina Legal, Universitat Autónoma de Barcelona, Cerdanyola del Vallés, Spain; 4 Red de Trastornos Adictivos, RETIC, Spain; 5 Centro de Investigación Biomédica en Red de Salud Mental, CIBERSAM G21, Spain; 6 Neuropharmacology, Universitat Pompeu Fabra, Barcelona, Spain; 7 Department of Psychiatry and Genetics, Washington University in St. Louis, Saint Louis, MO, United States

**Keywords:** Schizophrenia, Neurological Soft Signs, Temperament and character, Vulnerability markers, Personality

## Abstract

The heritability of schizophrenia and most personality traits has been well established, but the role of personality in susceptibility to schizophrenia remains uncertain. The aim of this study was to test for an association between personality traits and Neurological Soft Signs (NSS), a well-known biological marker of schizophrenia, in non-psychotic relatives of patients with schizophrenia. For this purpose, we evaluated the NSS scale and personality measured by the Temperament and Character inventory (TCI-R) in three groups of subjects: 29 patients with schizophrenia, 24 unaffected relatives and 37 controls. The results showed that patients with schizophrenia were more asocial (higher harm avoidance and lower reward dependence), more perseverative (higher persistence), and more schizotypal (lower self-directedness and cooperativeness, higher self-transcendence). The unaffected relatives showed higher harm avoidance, lower self-directedness and cooperativeness than the healthy controls. Higher NSS scores and sub-scores were found in patients and non-psychotic relatives compared with the controls. Among all the patients, total NSS scores were positively correlated with harm avoidance but negatively correlated with novelty seeking and persistence. Total NSS were also correlated with low scores on self-directedness and cooperativeness, which are indicators of personality disorder. Our results show that susceptibility to NSS and to schizophrenia are both related to individual differences in the temperament and character features in non-psychotic relatives of patients with schizophrenia. High harm avoidance, low persistence, low self-directedness and low cooperativeness contribute to both the risk of NSS and schizophrenia. These findings highlight the value of using both assessments to study high risk populations.

## Introduction

Although the etiology of schizophrenia is still largely unknown, the genetic basis of this disorder has been well established (Singh et al., 2014). The expression of susceptibility to schizophrenia is incomplete and variable, as shown by the non-psychotic status of most monozygotic co-twins of patients with schizophrenia, and the complex relationships of different sets of genes with distinct sets of clinical features (Arnedo et al., 2014). Fortunately, more subtle expressions of susceptibility to schizophrenia can be evaluated by studying neuropsychological markers of susceptibility, such as personality traits and neurological soft signs in the non-psychotic relatives of patients with schizophrenia (Smith et al., 2008).

Several studies have shown an association between schizophrenia and certain personality traits; however, the nature of this relationship is not clarified (Silberschmidt & Sponheim, 2008; Smith et al., 2008). Among the different models for studying personality, Cloninger’s model is the one with the most explicit neurobiological basis (Cloninger, Svrakic & Przybeck, 1993). This model suggests that a person’s temperament is heritable and regulated by neurotransmitters and brain circuits, which are involved in the pathophysiology of schizophrenia. Both temperament and character traits are equally heritable (Gillespie et al., 2003), but character is more shaped by sociocultural influences as it develops across a lifespan (Josefsson et al., 2013a; Josefsson et al., 2013b). Temperament consists of individual differences in behavioral conditioning of habits and skills, whereas character comprises of individual differences based on goals and values, which involve higher cognitive processes of semantic and autobiographical learning and memory (Silberschmidt & Sponheim, 2008; Van Schuerbeek et al., 2014). Environmental factors do impact on both temperament and character traits, however, these factors are more critical for the development of character than temperament. By using this model, patients with schizophrenia have shown a temperament and character profile that is distinct from the general population (Bora & Veznedaroglu, 2007; Glatt et al., 2006; Kurs, Farkas & Ritsner, 2005; Ritsner & Susser, 2004). Specifically, people with schizophrenia and their non-psychotic relatives are higher in the temperament of harm avoidance (i.e., more anxious and shy) and lower in the temperament of reward dependence (i.e., more detached and cold emotionally), so that they are more socially distant than controls. More recently, evidence has emerged showing that the dimensions of character are heritable and may also influence the risk of schizotypy (Silberschmidt & Sponheim, 2008). Specifically, people with schizophrenia and their non-psychotic relatives have the schizotypal character profile of low self-directedness (i.e., aimless and tending to blame others for their problems), low cooperativeness (i.e., suspicious and lacking in empathy), and high self-transcendence (i.e., prone to fantasy and magical thinking). Thus, Cloninger’s TCI provides a reliable way to quantify personality traits related to susceptibility to the schizophrenia spectrum.

The association between personality and schizophrenia has been reinforced by several studies that relate these personality traits with other abnormalities in schizophrenia. For example, the correlation between some dimensions of temperament and changes in monoaminergic activity has been postulated as the biological basis of schizophrenia (Ebstein, 2006; Mitsuyasu et al., 2001). In addition, an interaction has been observed between polymorphisms of these two systems that predicts the scores on harm avoidance (Benjamin et al., 2000).

Furthermore, several studies of schizophrenia have suggested an association between personality traits and other candidate markers of vulnerability. Specifically, the presence of schizotypal personality traits correlates with the presence of neurological soft signs (NSS) in relatives of patients with schizophrenia (Mechri et al., 2009; Mechri et al., 2010). Traditionally, NSS are defined as minor neurological abnormalities without a definite localization in the brain, including several clinical manifestations related to simple motor coordination, complex motor sequencing, sensory integration and disinhibition signs (Chan & Gottesman, 2008). Alterations in motor coordination and integration of stimuli are positively correlated with both the total scores and with the cognitive perceptive component of scales measuring schizotypy (Chan et al., 2010; Kaczorowski, Barrantes-Vidal & Kwapil, 2009). Thus, NSS have been suggested as markers of disease vulnerability, which are present prior to the start of treatment and are independent of illness state (as well as type of antipsychotic treatment) (Chan et al., 2010; Bombin, Arango & Buchanan, 2003), and NSS are correlated with structural and functional brain abnormalities related to schizophrenia (Mouchet-Mages et al., 2011; Zhao et al., 2014).

Interestingly, temperament and character features and NSS have been shown to aggregate in the relatives of schizophrenia patients (Krebs et al., 2000; Andreasen et al., 2005), supporting the view that both are likely to reflect genetic liability to schizophrenia. In addition, the distribution of NSS in schizophrenia, and in first-degree relatives, is consistent with the endophenotype criterion of familial association (Zhao et al., 2014). However, belonging to the same family could act as a confounding factor because it includes environmental influence and common genetic factors unrelated to the illness. In this respect, no studies are available comparing both NSS and personality in patients with schizophrenia and non-psychotic relatives.

The aim of this study was to investigate the association between personality traits, neurological soft signs and vulnerability to schizophrenia. Firstly, to determine whether personality traits could be vulnerability markers of schizophrenia, or if they are simply associated with the disease, we compared personality traits and neurological soft signs between patients, relatives and controls. Secondly, to establish whether those domains that showed differences between groups were significantly associated with known markers of disease vulnerability, correlations between personality traits and NSS were calculated for the entire population.

## Materials and Methods

### Subjects

A cross-sectional study was conducted on 29 patients with schizophrenia, 24 unaffected relatives of patients and 37 controls. This study was conducted at the Neuropsychiatry and Addictions Institute of the Parc de Salut Mar of Barcelona. The patients and the non-psychotic relatives were recruited from outpatient services of the same institution. Control subjects were recruited by announcements in the University and the Hospital. All participants lived in Spain for more than five years and were fluent Spanish speakers. The non-psychotic relatives were not from the same families of the patients included in the study, in order to avoid the effects of similar rearing that could induce potential similarities in temperament and character between patients and siblings. Considering that the total population was organized into three categories (patients, unaffected relatives and healthy controls), the participants were matched by gender and age.

The exclusion criteria included the presence of a substance dependence disorder (with the exception of nicotine dependence) according to DSM IV-TR (Diagnostic and Statistical Manual of Mental Disorders, Fourth Edition), the presence any other psychiatric disorder of axis I or II of DSM IV-TR as well as the personal history of severe somatic or neurological disorders. All subjects were between 25 and 50 years old and had an estimated IQ >80 measured by WAIS subscales (Digit, cubes, vocabulary, arithmetic, symbol search). The patients were diagnosed with schizophrenia from the medical record and confirmed by the Structured Clinical Interview for DSM Disorders. Unaffected relatives and healthy controls were evaluated as well. All the patients had a disease duration between 5 and 15 years, were treated with atypical antipsychotics, had never received electroconvulsive therapy and had been clinically stable for the last six months (all positive items of the PANSS positive subscale scoring 4 or lower). The non-psychotic relatives were from the same mother and father of a patient with a diagnosis of schizophrenia, according to DSM IV-TR. Control subjects and their first and second degree relatives had to be free of any axis I disorders. The study was approved by the ethics committee of the CEIC-Parc de Salut Mar Hospital (2011/4141/I). All subjects gave informed written consent and were assured of the confidentiality of the data being collected.

### Experimental procedure

Basic socio-biographical data were collected from the medical history. This data included years of education, socio-economic level, psychiatric and medical history, years from disease onset, administered treatment and psychiatric history of first degree relatives. Patients were clinically assessed using the Positive and Negative Syndrome Scale (PANSS) (Peralta & Cuesta, 1994) and the overall functioning of the subjects was assessed using the Global Adaptive Functioning (GAF) (Jones et al., 1995).

All subjects were assessed with the Spanish version of the Temperament and Character Inventory (TCI-R) (Gutiérrez-Zotes et al., 0000) and the Neurological Soft Signs Scale (Krebs et al., 2000). Temperament is comprised of novelty seeking (i.e., impulsive, exploratory), harm avoidance (i.e., anxious, shy), reward dependence (i.e., sentimental, approval-seeking) and persistence (i.e., determined, ambitious). Character is comprised of self-directedness (i.e., responsible, purposeful), cooperativeness (i.e., helpful, empathic) and self-transcendence (i.e., imaginative, self-forgetful). The TCI-R sub-scores for each of the seven dimensions were calculated.

The NSS scale is composed of 23 items, rated from 0 to 3, and regrouped in five consistent factors: Motor coordination (hand dysrhythmia, finger opposition, fist edge–palm, foot dysrhythmia, alternative movements: foot speed, alternative movements: hand speed, standing heel-to-toe), Motor integration (Romberg, apraxia, tandem walk, finger-to-nose, gait, tongue protrusion), Sensory integration (stereognosia, hand–face, constructive apraxia, graphesthesia, right-left recognition), Quality of lateralization (right-left confusion, lateral preference, right-left asymmetry) and Involuntary movements (abnormal movement and posture, mirror movements).

The NSS total score and sub-scores for each of the factors were calculated. Two assessors (LG and FP) were trained to perform the neurological assessment. The inter-rater reliability of the assessment of NSS was established by the two assessors and jointly examined 20 independent subjects. The intra-class correlation coefficient (SPSS: two way Mixed Effect Model, confidence interval = 95%) was 0.90 [0.77–0.95].

### Statistical analysis

First, univariate analyses of the sociodemographic data were performed. Differences in age and years of education were determined with the Analysis of Variance (ANOVA), and the *chi* square test was applied for gender differences. As there were statistical differences in years of education, it was added as a covariate in the rest of the analyses.

Temperament, character and NSS scores were analyzed with the Levene test. Then, to study differences between groups, and depending on the results on Levene tests, the analysis was performed with ANOVAs followed by the Bonferroni post-hoc test or a Kruskall–Wallis test followed by Mann–Whitney U test, adding years of education as a covariate. Pearson correlations were performed using the entire population between the total NSS scores and sub-scores for each temperament and character domains, adding years of education as a covariate. Accepting an alpha risk of 0.05 and a beta risk of 0.2 in a two-sided test, 23 subjects are necessary in every group to recognize as statistically significant a difference greater than or equal to 1 unit. The common standard deviation is assumed to be 1.2. It has been anticipated a drop-out rate of 0%.

## Results

### Demographic characteristics

No significant differences between groups were observed in terms of age or gender; although patients with schizophrenia and non-psychotic relatives showed significantly less years of education than controls (Table 1).

**Table 1:** Demographic characteristics in controls, non-psychotic relatives and patients with schizophrenia.

	Controls	Non-psychotic relatives	Patients	*p*
	*n* = 37	*n* = 24	*n* = 29	
Mean Age (years) ± SD	36.78 ± 7.61	40.92 ± 10.32	37.97 ± 7.13	0.165
Gender (M/F)	17/20	11/13	16/13	0.713
Mean years of education (years) ± SD	12.89 ± 1.76	11.50 ± 2.65	10.00 ± 2.80	<0.05^∗^

### Temperament scores (TCI-R)

**Figure 1:**
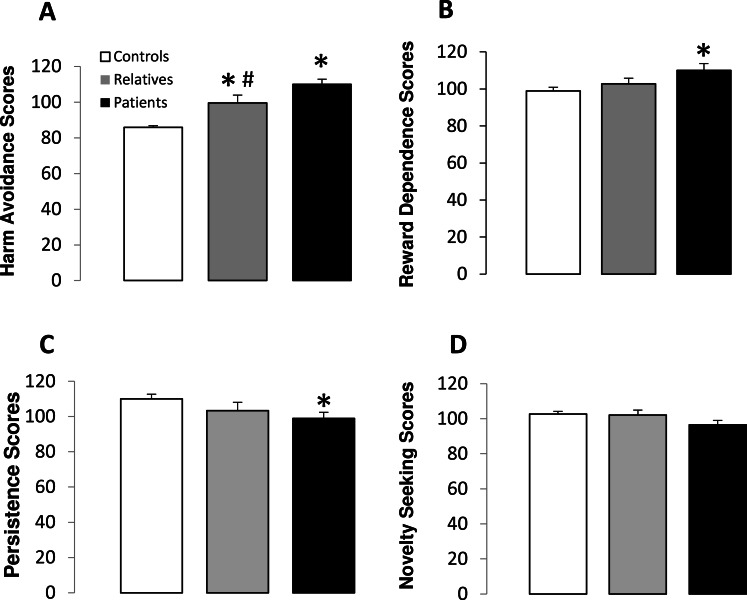
Temperament scores in controls, non-psychotic relatives and patients with schizophrenia. Harm avoidance (A), reward dependence (B), persistence (C) and novelty seeking (D) scores. The data are represented as mean + SD. ^∗^*p* < 0.05 vs. controls; #*p* < 0.05 vs. relatives.

Table 2 shows the scores obtained for each temperament dimension in controls, non-psychotic relatives and patients. Harm Avoidance scores were significantly different between the groups (*F*(2,88) = 13.10, *p* < 0.001) (Fig. 1). Subsequent post-hoc analysis revealed that patients with schizophrenia and non-psychotic relatives obtained significantly higher scores on harm avoidance than controls, and patients showed significantly higher scores than relatives (Fig. 1). In addition, significant differences between the groups were observed in reward dependence (*F*(2,88) = 3.15, *p* < 0.05) and persistence (*F*(2,88) = 3.83, *p* < 0.05) scores. The post-hoc test revealed that patients obtained significantly lower reward dependence scores than controls and both patients and non-psychotic relatives had lower persistence scores than controls. No significant differences between groups were observed for novelty seeking scores (Fig. 1).

**Table 2:** Temperament and character scores in controls, non-psychotic relatives and patients with schizophrenia.

		Controls	Non-psychotic relatives	Patients	*F*	*p*
		*n* = 37	*n* = 24	*n* = 29		
Temperament	Harm avoidance (mean ± SEM)	86.18 ± 1.85	99.38 ± 4.49	109.21 ± 2.97	13.10	<0.01^∗^
	Reward dependence (mean ± SEM)	109.36 ± 2.03	101.38 ± 3.13	99.79 ± 3.66	3.15	<0.05^∗^
	Novelty seeking (mean ± SEM)	102.39 ± 1.55	102.81 ± 2.88	97.7 ± 2.43	1.29	0.27
	Persistence (mean ± SEM)	113.91 ± 2.63	103.14 ± 4.75	100.58 ± 3.50	3.83	<0.05^∗^
Character	Self-directedness (mean ± SEM)	159.62 ± 2.87	141.14 ± 4.64	134.67 ± 4.40	10.11	<0.01^∗^
	Cooperativeness (mean ± SEM)	147.17 ± 2.24	137.05 ± 2.57	133.12 ± 3.86	5.59	<0.05∗
	Self-Transcendence (mean ± SEM)	54.30 ± 2.56	58.76 ± 2.82	66.25 ± 3.99	3.63	<0.05^∗^

### Character scores (TCI-R)

**Figure 2:**
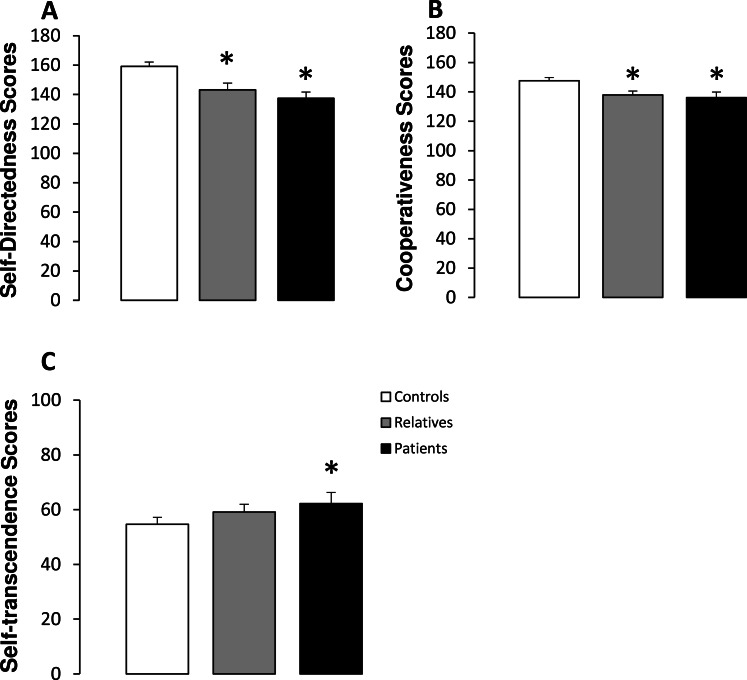
Character scores in controls, non-psychotic relatives and patients with schizophrenia. Self-directedness (A), cooperativeness (B) and self-transcendence (C) scores. The data are represented as mean + SD. ^∗^*p* < 0.05 vs. controls.

Table 2 shows the scores obtained for each character dimension in controls, non-psychotic relatives and patients. Significant differences between groups were observed in self-directedness, cooperativeness and self-transcendence scores. A subsequent subgroups analysis revealed that both patients and relatives obtained significantly lower scores on self-directedness and cooperativeness than the controls (Fig. 2). In addition, no significant differences were observed in self-directedness or cooperativeness scores between patients and relatives. Finally, significantly higher self-transcendence scores were observed in patients with schizophrenia than in the controls (Fig. 2).

### Neurological soft signs scores

**Figure 3:**
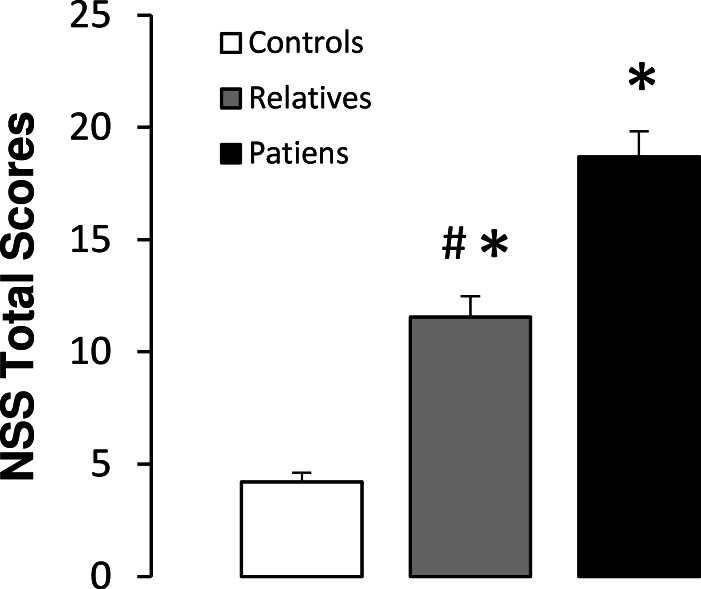
Total neurological soft signs (NSS) scores in controls, non-psychotic relatives and patients with schizophrenia. The data are represented as mean + SD. ^∗^*p* < 0.05 vs. controls; #*p* < 0.05 vs. relatives.

Significant differences between groups were observed for the total NSS scores (*F*(2,88) = 41.98, *p* < 0.01). A subsequent post-hoc analysis revealed significantly higher NSS scores in both non-psychotic relatives and patients, compared with the control subjects. In addition, patients showed higher total NSS scores than non-psychotic relatives (Fig. 3). Scores obtained in each NSS domain for the three groups are shown in Table 3. Significant differences between groups were observed for each of the NSS sub-scores. Post-hoc analyses revealed significantly higher scores in motor coordination and involuntary movements in patients and relatives, as compared with the controls. In addition, patients showed higher scores than relatives in both of these NSS sub-scores. With respect to motor integration and quality of lateralization, patients and relatives also showed higher scores than control subjects, while no significant differences were observed between patients and relatives. For sensory integration, higher scores were observed only in patients compared with the control group (Fig. 3).

**Table 3:** NSS scores in controls, non-psychotic relatives and patients with schizophrenia.

Neurological soft sign scores	Controls	Non-psychotic relatives	Patients	*F*	*P*
	*n* = 37	*n* = 24	*n* = 29		
Motor coordination (mean ± SEM)	0.71 ± 0.18	1.65 ± 0.34	3.13 ± 0.37	15.32	<0.001^∗^
Sensory integration (mean ± SEM)	1.13 ± 0.15	1.65 ± 0.18	2.57 ± 0.38	6.31	<0.001^∗^
Motor integration (mean ± SEM)	1.32 ± 0.11	4.85 ± 0.33	4.52 ± 0.33	36.29	<0.001^∗^
Quality of lateralization (mean ± SEM)	0.29 ± 0.09	0.95 ± 0.34	0.73 ± 0.17	4.20	<0.01^∗^
Involuntary movement (mean ± SEM)	0.94 ± 0.15	1.25 ± 0.18	2.78 ± 0.40	13.03	<0.001^∗^

### Correlations between NSS and TCI-R scores

Table 4 shows the Pearson coefficients obtained for correlations between NSS scores and temperament and character scores for the entire population studied. In terms of temperament, total NSS scores were positively correlated with harm avoidance, while a negative correlation was observed between total NSS, novelty seeking and persistence scores. When each temperament dimension was analyzed separately, harm avoidance scores correlated significantly with sensory integration, motor coordination and motor integration scores. For persistence, significant negative correlations were observed with motor coordination, sensory integration, motor integration and involuntary movements. Finally, a positive correlation was observed between reward dependence and involuntary movements. Novelty seeking scores were negatively correlated with sensory integration. With regards to character, total NSS scores were negatively correlated with self-directedness and cooperativeness. For the individual character domains, self-directedness was negatively correlated with motor coordination and motor integration scores, while cooperativeness was negatively correlated with sensory integration, motor integration and motor coordination scores. No significant correlations were observed between self-transcendence and total NSS scores, although a positive correlation was present with motor coordination.

**Table 4:** Correlations coefficients between NSS and temperament features and between NSS and character traits in controls, non-psychotic relatives and patients with schizophrenia.

	Total NSS	Sensory integration	Motor coordination	Motor integration	Quality lateralization	Involuntary movement
Harm avoidance	0.95^∗^	0.38^∗^	0.35^∗^^∗^	0.48^∗^	0.03	0.16
Reward dependence	−0.12	−0.12	−0.16	−0.15	0.10	0.25^∗^
Novelty seeking	−0.40^∗^	−0.84^∗^	−0.22	−0.15	−0.29^∗^	−0.15
Persistence	−0.95^∗^	−0.43^∗^	0.29*	−0.43^∗^	−0.08	−0.40^∗^
Self-directedness	−0.80^∗^	−0.18	−0.39^∗^	−0.40^∗^	0.03	−0.08
Cooperativeness	−0.55^∗^	−0.22^∗^	−0.32^∗^	−0.23^∗^	−0.01	−0.13
Self-transcendence	0.19	0.07	0.27^∗^	0.20	−0.01	−0.01

## Discussion

The major finding in this study was that patients with schizophrenia and non-psychotic relatives display a unique profile of temperament and character that correlates with alterations in NSS. Comparing personality traits and NSS between groups, both patients with schizophrenia and non-psychotic relatives obtained significantly higher scores on harm avoidance than controls, and patients showed significantly higher scores than relatives. Also, patients and non-psychotic relatives had lower persistence, self-directedness, and cooperativeness scores than controls. In addition, no significant differences were observed in self-directedness or cooperativeness scores between patients and relatives. Finally, significantly higher self-transcendence scores were observed in patients with schizophrenia, compared to controls.

Our results reveal an association between these hypothesized vulnerability markers, as temperament (especially harm avoidance, reward dependence and persistence) and character (especially self-directedness and cooperativeness) correlated with the presence of NSS in the entire sample.

Studies in non-psychotic relatives have been essential to uncover new vulnerability biomarkers of schizophrenia. In this sense, several studies have provided evidence showing that particular personality features could be considered as possible schizophrenia-related endophenotypes (Smith et al., 2008). In this study, it was found that non-psychotic relatives had significantly higher harm avoidance scores compared with the controls, but lower scores than patients with schizophrenia. In agreement with our data, Smith et al. (2008) found higher harm avoidance scores in siblings of patients with schizophrenia than in controls subjects, and another study reported that siblings are positioned between controls and patients with schizophrenia, in terms of temperament profile (Calvó de Padilla et al., 2006). In contrast, Bora & Veznedaroglu (2007) did not find differences in temperament between relatives of schizophrenic patients and the controls, although they did observe differences in harm avoidance between controls and relatives with high schizotypy. Together, these studies support the idea that high levels of harm avoidance may be associated with genetic vulnerability to schizophrenia, which, in turn, will interact with environmental and neurobiological influences to determine the expression of the disease. According to Kim et al. (2011) and Hansenne et al. (2003), harm avoidance has been associated with D2/3 receptor availability in the associative and sensorimotor subdivisions of the striatum and high Mismatch Negativity and hypervigilant fear perception, suggesting abnormal sensory gating of aversive stimuli as a vulnerability variable in schizophrenia. Furthermore, a locus on chromosome 8p21 associated to schizophrenia showed a linkage to harm avoidance (Zohar et al., 2003).

With regards to character, it was found that, similar to patients; non-psychotic relatives had significantly lower self-directedness and cooperativeness scores when compared to controls. Other studies have reported lower levels of self-directedness and cooperativeness in siblings with high schizotypy as compared to controls, and high levels were observed in siblings with low schizotypy (Bora & Veznedaroglu, 2007). One important aspect of the data in this study is that even though the non-psychotic relatives that participated in this study did not have familial ties to the patients with schizophrenia, they showed similar low levels of self-directedness and cooperativeness. It is well known that character is influenced more by environmental factors than temperament (Josefsson et al., 2013a; Josefsson et al., 2013b). However, the data in this study agrees with other studies, such as Gillespie et al., (2003); Josefsson et al., (2013a); Josefsson et al., (2013b), showing that character may also have a genetic component. Self-transcendence was higher in patients than in the controls subject, but not in relatives. These results are in agreement with other studies reporting elevated self-transcendence in patients (Glatt et al., 2006; Smith et al., 2008). In contrast, Calvó de Padilla et al. (2006) found lower self-transcendence and cooperativeness in the relatives of patients with schizophrenia with respect to the controls. The discrepancies between studies could be due to the fact that the population used in the Calvo and Padilla study was an indigenous community living in a rural environment and not in an urban environment.

In accordance with previous studies, we found lower levels of persistence and reward dependence only in patients with schizophrenia as compared to controls. These findings endorse the hypothesis stating that high harm avoidance, low persistence and low reward dependence constitutes a temperament profile leading to social detachment, perseveration and schizotypy, when combined with a disorganized character profile that impairs emotional regulation (Smith et al., 2008; Bora & Veznedaroglu, 2007).

As reported previously in patients with schizophrenia (Bombin, Arango & Buchanan, 2003; Chen et al., 2005; Aksoy-Poyraz et al., 2011) and in non-psychotic relatives (Gourion et al., 2004; Mechri et al., 2010), we found higher NSS in both groups as compared with the controls, confirming the hypothesis that NSS is a vulnerability marker for schizophrenia. In addition, these results agree with the idea that NSS segregate with the illness and may be a valid and useful endophenotype (Chan et al., 2010). The association between personality characteristics and NSS has been studied separately in siblings, or in patients with schizophrenia, but there are no prior studies correlating NSS with personality traits in patients with schizophrenia, non-psychotic relatives and controls. Our correlational analysis, including all three groups, showed that subjects with higher NSS scores exhibited higher harm-avoidance and persistence scores, while they exhibited lower self-directness and cooperativeness. Two related studies have evaluated the association between NSS and schizotypal personality traits with contradictory results. Thus, Mechri et al. (2010), using the Schizotypal Personality Questionnaire (SPQ), showed that the overall NSS score was correlated with the presence of schizotypal traits in both non-psychotic siblings and controls, while no association was found between NSS and schizotypal dimensions in relatives of patients with schizophrenia, when the SPQ test was used (Bollini et al., 2007). The differences observed between these two studies, as well as the present work, could be due to the fact that they used a personality assessment tool based on outdated DSM III criteria. In this respect, one of the strengths of this study is the use of the TCI-R scale, which is a comprehensive personality questionnaire that has been extensively validated in clinical practice and research (Fassino et al., 2013; Fresán et al., 2015). One of the advantages of the TCI-R is that it explores normal and pathological personalities in subjects with mental disorders and also in the general population (Cloninger et al., 2012; Josefsson et al., 2011; De Fruyt et al., 2006). Another advantage is that temperament and character domains have been associated with structural and functional changes in the brain (Laricchiuta et al., 2014; Lei et al., 2014; Tuominen et al., 2013), and have been related to specific chromosomal regions (Serretti et al., 2008; Zohar et al., 2003) supporting the neurobiological substrate for this personality model (Yang et al., 2015). Another strength of the study is that the relatives of patients with schizophrenia had no familial ties to the patients used, thus decreasing the possibility that similar rearing would confound the results.

Finally, several limitations of the study are acknowledged. The first is the small sample size used, even though the TCI-R scores and NSS scores were similar to those reported in larger samples in the literature (Smith et al., 2008; Mechri et al., 2010). The second limitation is the use of an estimate of IQ values as a selection criterion, but not as a covariate in the analysis. This issue may have been a potential confounding factor, since IQ has been previously associated with personality and with NSS.

In conclusion, these results showed that patients with schizophrenia were more asocial (higher harm avoidance and lower reward dependence), more perseverative (higher persistence) and more schizotypal (lower self-directedness and cooperativeness, higher self-transcendence). In the group analysis, we found significant changes in personality traits in relatives of patients with schizophrenia. Indeed, non-psychotic relatives showed higher harm avoidance, lower self-directness and lower cooperativeness when compared to control subjects. Interestingly, all three items were correlated with total NSS scores. Thus, a positive correlation was observed between higher harm avoidance and total NSS, and negative correlations were found between lower self-directedness and lower cooperativeness with total NSS. These findings lend support to the idea that such personality traits could be potential vulnerability markers for schizophrenia. These vulnerability markers are likely to be useful tools in the prospective studies of high-risk populations.

## Supplemental Information

10.7717/peerj.1651/supp-1 Data S1 Raw Data

## Additional Information and Declarations

### Data Availability

The following information was supplied regarding data availability: The raw data has been supplied as Data S1.

## References

[ref-1] Aksoy-Poyraz C, Poyraz BÇ, Turan S, Arikan MK (2011). Minor physical anomalies and neurological soft signs in patients with schizophrenia and their siblings. Psychiatry Research.

[ref-2] Andreasen NC, Carpenter WT, Kane JM, Lasser RA, Marder SR, Weinberger DR (2005). Remission in schizophrenia: proposed criteria and rationale for consensus. American Journal of Psychiatry.

[ref-3] Arnedo J, Svrakic DM, Del Val C, Romero-Zaliz R, Hernández-Cuervo H, Fanous AH, Pato MT, Pato CN, De Erausquin GA, Cloninger CR, Zwir I (2014). Uncovering the hidden risk architecture of the schizophrenias: confirmation in three independent genome-wide association studies. The American Journal of Psychiatry.

[ref-4] Benjamin J, Osher Y, Kotler M, Gritsenko I, Nemanov L, Belmaker RH, Ebstein RP (2000). Association between tridimensional personality questionnaire (TPQ) traits and three functional polymorphisms: dopamine receptor D4 (DRD4), serotonin transporter promoter region (5-HTTLPR) and catechol O-methyltransferase (COMT). Molecular Psychiatry.

[ref-5] Bollini AM, Compton MT, Esterberg ML, Rutland J, Chien VH, Walker EF (2007). Associations between schizotypal features and indicators of neurological and morphological abnormalities. Schizophrenia Research.

[ref-6] Bombin I, Arango C, Buchanan RW (2003). Assessment tools for soft signs-a review of the major scales used in research on neurological soft signs in schizophrenia, with recommendations for future directions of soft sign assessment. Psychiatric Annals.

[ref-7] Bora E, Veznedaroglu B (2007). Temperament and character dimensions of the relatives of schizophrenia patients and controls: the relationship between schizotypal features and personality. European Psychiatry.

[ref-8] Calvó de Padilla M, Padilla E, González Alemán G, Bourdieu M, Guerrero G, Strejilevich S, Escobar JI, Svrakic N, Cloninger CR, De Erausquin GA (2006). Temperament traits associated with risk of schizophrenia in an indigenous population of Argentina. Schizophrenia Research.

[ref-9] Chan RCK, Gottesman II (2008). Neurological soft signs as candidate endophenotypes for schizophrenia: a shooting star or a Northern star?. Neuroscience and Biobehavioral Reviews.

[ref-10] Chan RCK, Wang Y, Zhao Q, Yan C, Xu T, Gong Q-Y, Manschreck TC (2010). Neurological soft signs in individuals with schizotypal personality features. The Australian and New Zealand Journal of Psychiatry.

[ref-11] Chen EYH, Hui CLM, Chan RCK, Dunn ELW, Miao MYK, Yeung WS, Wong C-K, Chan W-F, Tang WN (2005). A 3-year prospective study of neurological soft signs in first-episode schizophrenia. Schizophrenia Research.

[ref-12] Cloninger CR, Svrakic DM, Przybeck TR (1993). A psychobiological model of temperament and character. Archives of General Psychiatry.

[ref-13] Cloninger CR, Zohar AH, Hirschmann S, Dahan D (2012). The psychological costs and benefits of being highly persistent: personality profiles distinguish mood disorders from anxiety disorders. Journal of Affective Disorders.

[ref-14] De Fruyt F, De Clercq BJ, Van Wiele L De, Van Heeringen K (2006). The validity of cloninger’s psychobiological model versus the five-factor model to predict DSM-IV personality disorders in a heterogeneous psychiatric sample: domain facet and residualized facet descriptions. Journal of Personality.

[ref-15] Ebstein RP (2006). The molecular genetic architecture of human personality: beyond self-report questionnaires. Molecular Psychiatry.

[ref-16] Fassino S, Amianto F, Sobrero C, Abbate Daga G (2013). Does it exist a personality core of mental illness? A systematic review on core psychobiological personality traits in mental disorders. Panminerva Medica.

[ref-17] Fresán A, León-Ortiz P, Robles-García R, Azcárraga M, Guizar D, Reyes-Madrigal F, Tovilla-Zárate CA, De la Fuente-Sandoval C (2015). Personality features in ultra-high risk for psychosis: a comparative study with schizophrenia and control subjects using the Temperament and Character Inventory-Revised (TCI-R). Journal of Psychiatric Research.

[ref-18] Gillespie NA, Cloninger CR, Heath AC, Martin NG (2003). The genetic and environmental relationship between Cloninger’s dimensions of temperament and character. Personality and Individual Differences.

[ref-19] Glatt SJ, Stone WS, Faraone SV, Seidman LJ, Tsuang MT (2006). Psychopathology, personality traits and social development of young first-degree relatives of patients with schizophrenia. The British Journal of Psychiatry: The Journal of Mental Science.

[ref-20] Gourion D, Goldberger C, Olie JP, Lôo H, Krebs MO (2004). Neurological and morphological anomalies and the genetic liability to schizophrenia: a composite phenotype. Schizophrenia Research.

[ref-21] Gutiérrez-Zotes JA, Bayón C, Montserrat C, Valero J, Labad A, Cloninger CR, Fernández-Aranda F Temperament and Character Inventory Revised (TCI-R). Standardization and normative data in a general population sample. Actas Españolas de Psiquiatría.

[ref-22] Hansenne M, Pinto E, Scantamburlo G, Couvreur A, Reggers J, Fuchs S, Pitchot W, Ansseau M (2003). Mismatch negativity is not correlated with neuroendocrine indicators of catecholaminergic activity in healthy subjects. Human Psychopharmacology.

[ref-23] Jones SH, Thornicroft G, Coffey M, Dunn G (1995). A brief mental health outcome scale-reliability and validity of the Global Assessment of Functioning (GAF). The British Journal of Psychiatry: The Journal of Mental Science.

[ref-24] Josefsson K, Cloninger CR, Hintsanen M, Jokela M, Pulkki-Råback L, Keltikangas-Järvinen L (2011). Associations of personality profiles with various aspects of well-being: a population-based study. Journal of Affective Disorders.

[ref-25] Josefsson K, Jokela M, Cloninger CR, Hintsanen M, Salo J, Hintsa T, Pulkki-Råback L, Keltikangas-Järvinen L (2013a). Maturity and change in personality: developmental trends of temperament and character in adulthood. Development and Psychopathology.

[ref-26] Josefsson K, Jokela M, Hintsanen M, Robert Cloninger C, Pulkki-Råback L, Merjonen P, Hutri-Kähönen N, Keltikangas-Järvinen L (2013b). Parental care-giving and home environment predicting offspring’s temperament and character traits after 18 years. Psychiatry Research.

[ref-27] Kaczorowski JA, Barrantes-Vidal N, Kwapil TR (2009). Neurological soft signs in psychometrically identified schizotypy. Schizophrenia Research.

[ref-28] Kim JH, Son YD, Kim HK, Lee SY, Cho SE, Kim YB, Cho ZH (2011). Association of harm avoidance with dopamine D2/3 receptor availability in striatal subdivisions: a high resolution PET study. Biological Psychology.

[ref-29] Krebs MO, Gut-Fayand A, Bourdel MC, Dischamp J, Olié JP (2000). Validation and factorial structure of a standardized neurological examination assessing neurological soft signs in schizophrenia. Schizophrenia Research.

[ref-30] Kurs R, Farkas H, Ritsner M (2005). Quality of life and temperament factors in schizophrenia: comparative study of patients, their siblings and controls. Quality of Life Research.

[ref-31] Laricchiuta D, Petrosini L, Piras F, Macci E, Cutuli D, Chiapponi C, Cerasa A, Picerni E, Caltagirone C, Girardi P, Tamorri SM, Spalletta G (2014). Linking novelty seeking and harm avoidance personality traits to cerebellar volumes. Human Brain Mapping.

[ref-32] Lei X, Chen C, Xue F, He Q, Chen C, Liu Q, Moyzis RK, Xue G, Cao Z, Li J, Li H, Zhu B, Liu Y, Hsu ASC, Li J, Dong Q (2014). Fiber connectivity between the striatum and cortical and subcortical regions is associated with temperaments in Chinese males. NeuroImage.

[ref-33] Mechri A, Bourdel MC, Slama H, Gourion D, Gaha L, Krebs MO (2009). Neurological soft signs in patients with schizophrenia and their unaffected siblings: frequency and correlates in two ethnic and socioeconomic distinct populations. European Archives of Psychiatry and Clinical Neuroscience.

[ref-34] Mechri A, Gassab L, Slama H, Gaha L, Saoud M, Krebs MO (2010). Neurological soft signs and schizotypal dimensions in unaffected siblings of patients with schizophrenia. Psychiatry Research.

[ref-35] Mitsuyasu H, Hirata N, Sakai Y, Shibata H, Takeda Y, Ninomiya H, Kawasaki H, Tashiro N, Fukumaki Y (2001). Association analysis of polymorphisms in the upstream region of the human dopamine D4 receptor gene (DRD4) with schizophrenia and personality traits. Journal of Human Genetics.

[ref-36] Mouchet-Mages S, Rodrigo S, Cachia A, Mouaffak F, Olie JP, Meder JF, Oppenheim C, Krebs MO (2011). Correlations of cerebello-thalamo-prefrontal structure and neurological soft signs in patients with first-episode psychosis. Acta Psychiatrica Scandinavica.

[ref-37] Peralta V, Cuesta MJ (1994). Psychometric properties of the positive and negative syndrome scale (PANSS) in schizophrenia. Psychiatry Research.

[ref-38] Ritsner M, Susser E (2004). Temperament types are associated with weak self-construct, elevated distress and emotion-oriented coping in schizophrenia: evidence for a complex vulnerability marker?. Psychiatry Research.

[ref-39] Serretti A, Benedetti F, Mandelli L, Calati R, Caneva B, Lorenzi C, Fontana V, Colombo C, Smeraldi E (2008). Association between GSK-3?? -50T/C polymorphism and personality and psychotic symptoms in mood disorders. Psychiatry Research.

[ref-40] Silberschmidt AL, Sponheim SR (2008). Personality in relation to genetic liability for schizophrenia and bipolar disorder: differential associations with the COMT Val108/158Met polymorphism. Schizophrenia Research.

[ref-41] Singh S, Kumar A, Agarwal S, Phadke SR, Jaiswal Y (2014). Genetic insight of schizophrenia: past and future perspectives. Gene.

[ref-42] Smith MJ, Cloninger CR, Harms MP, Csernansky JG (2008). Temperament and character as schizophrenia-related endophenotypes in non-psychotic siblings. Schizophrenia Research.

[ref-43] Tuominen L, Salo J, Hirvonen J, Någren K, Laine P, Melartin T, Isometsä E, Viikari J, Cloninger CR, Raitakari O, Hietala J, Keltikangas-Järvinen L (2013). Temperament, character and serotonin activity in the human brain: a positron emission tomography study based on a general population cohort. Psychological Medicine.

[ref-44] Van Schuerbeek P, Baeken C, Luypaert R, De Raedt R, De Mey J (2014). Does the amygdala response correlate with the personality trait harm avoidance while evaluating emotional stimuli explicitly?. Behavioral and Brain Functions.

[ref-45] Yang S, Sung J, Kim J-H, Song Y-M, Lee K, Kim H-N, Kim H-L, Cloninger CR (2015). Some personality traits converge gradually by long-term partnership through the lifecourse—genetic and environmental structure of Cloninger’s temperament and character dimensions. Journal of Psychiatric Research.

[ref-46] Zhao Q, Li Z, Huang J, Yan C, Dazzan P, Pantelis C, Cheung EFC, Lui SSY, Chan RCK (2014). Neurological soft signs are not soft in brain structure and functional networks: evidence from ALE meta-analysis. Schizophrenia Bulletin.

[ref-47] Zohar AH, Dina C, Rosolio N, Osher Y, Gritsenko I, Bachner-Melman R, Benjamin J, Belmaker RH, Ebstein RP (2003). Tridimensional personality questionnaire trait of harm avoidance (anxiety proneness) is linked to a locus on chromosome 8p21. American Journal of Medical Genetics. Part B, Neuropsychiatric Genetics: The Official Publication of the International Society of Psychiatric Genetics.

