# Supplementary material for: Association between neurological soft signs, temperament and character in patients with schizophrenia and non-psychotic relatives
Source: PeerJ. 2016 Apr 26;4:e1651. doi: 10.7717/peerj.1651 (PMC4860298; doi:10.7717/peerj.1651)
Supplement: Data S1 [file peerj-04-1651-s001.pdf]

	NS	HA	RD	P	SD	C	
P1	119	115	93	88	121	122	
P2	81	103	129	93	170	164	
P3	83	125	87	91	145	158	
P6	92	111	82	84	157	133	
P7	76	115	109	132	124	130	
P8	98	128	113	119	138	145	
P9	92	85	101	104	170	135	
P10	97	124	116	96	127	148	
P11	99	117	116	105	129	144	
P12	112	101	97	101	135	129	
P13	119	93	98	129	135	123	
P14	94	125	104	89	125	152	
P15	102	98	90	134	105	116	
P16	86	127	85	95	108	112	
P17	102	122	130	104	119	143	
P18	82	101	142	119	180	172	
P19	98	107	76	70	156	138	
P20	81	103	129	93	170	164	
P21	83	125	87	91	145	158	
P22	102	100	88	98	122	123	
P23	109	143	70	72	100	120	
P24	66	75	126	111	188	167	
P25	94	125	104	89	125	152	
P26	111	81	78	107	132	96	
P27	98	107	76	70	156	138	
P28	102	115	72	68	101	87	
P29	108	89	96	121	124	120	
P30	115	121	97	84	121	118	
P31	98	107	76	70	156	138	
F1	85	95	128	112	166	151	
F2	113	122	80	73	121	139	
F3	109	86	78	145	139	110	
F4	85	92	118	84	165	152	
F5	99	115	103	83	158	146	
F6	81	84	86	128	133	130	
F7	81	105	96	87	141	134	
F8	103	120	124	102	150	145	
F9	102	96	110	122	157	136	
F10	106	56	114	94	164	163	
F11	138	84	117	120	145	124	
F12	95	102	101	95	164	147	
F13	102	96	110	122	157	136	
F14	117	92	101	95	154	140	
F15	98	118	93	88	100	120	
F16	125	84	116	137	154	153	
F17	110	54	96	147	159	138	
F18	85	92	118	84	165	152	
F19	92	144	81	48	79	132	
F20	99	140	107	92	135	137	
F21	101	81	96	108	148	136	
F22	99	108	97	104	127	129	
F23	121	109	87	102	105	116	
F24	103	120	124	102	150	145	
C1	121	87	108	119	144	132	
C2	113	77	115	145	158	154	

C3	94	91	86	114	131	135
C4	107	77	109	109	176	151
C5	94	89	108	126	159	153
C6	91	84	124	108	181	171
C8	108	64	124	133	165	162
C9	108	81	98	98	133	134
C10	101	87	109	113	154	140
C11	99	85	108	110	177	154
C12	104	71	128	139	188	155
C13	104	91	107	110	156	155
C14	129	80	129	143	167	163
C17	99	85	104	111	178	150
C18	100	103	99	110	146	123
C19	94	89	108	126	159	153
C20	119	82	115	113	150	138
C21	113	88	95	73	152	129
C22	100	77	110	99	170	140
C24	117	94	85	102	130	119
C25	108	66	123	117	190	164
C26	91	76	108	113	158	143
C27	107	96	116	128	145	156
C28	92	83	132	135	183	159
C29	101	95	87	101	147	147
C30	101	104	87	86	136	128
C31	89	81	107	118	180	158
C32	101	75	115	113	167	130
C33	108	105	101	93	150	131
C34	101	92	113	127	132	133
C35	99	100	120	108	170	150
C36	97	75	119	121	175	161
C37	90	69	110	94	184	173
C38	91	99	105	132	167	158
C39	97	102	115	118	138	149
C40	98	104	113	119	139	149
C41	112	71	133	140	156	160

ST	SI	MI	MC	IM	QL	NSS	
	77	6	3	5	4	2	22
	45	8	4	7	6	2	29
	46	2	3	7	2	0	10
	37	2	0	3	1	2	11
	99	6	3	5	1	3	24
	71	3	2	6	0	1	13
	41	2	0	3	1	2	12
	99	3	5	5	1	0	13
	71	1	4	5	4	1	23
	69	1	3	8	1	1	17
	69	0	3	2	0	0	5
	57	2	5	7	2	1	19
	73	1	4	5	4	1	23
	97	4	6	6	5	0	19
	91	3	2	5	2	0	12
	38	2	5	3	5	2	16
	34	5	5	5	7	0	24
	45	8	4	7	6	2	29
	46	3	3	6	2	0	15
	81	4	5	5	4	2	24
	50	2	5	5	3	0	15
	42	4	5	5	4	2	24
	57	2	5	7	2	1	19
	70	1	4	5	4	1	23
	34	5	5	5	7	0	24
	89	5	7	4	3	1	23
	88	2	0	3	1	2	11
	56	4	6	6	5	0	19
	34	5	5	5	7	0	24
	50	2	4	6	2	5	21
	40	1	1	3	1	0	6
	46	2	0	3	0	0	6
	48	3	2	4	2	3	16
	41	1	0	4	0	0	5
	64	2	0	5	1	0	8
	77	2	3	7	1	2	16
	52	2	4	5	2	0	13
	86	0	3	8	1	0	12
	54	0	0	5	1	1	9
	82	3	0	3	4	0	10
	44	1	0	7	2	1	12
	86	0	3	8	1	0	12
	48	2	0	2	2	1	9
	54	2	4	5	1	0	12
	55	1	2	5	1	0	9
	71	1	1	6	0	0	8
	48	3	2	4	2	3	16
	63	2	0	3	0	0	5
	58	1	1	4	1	0	9
	63	2	4	5	1	0	12
	66	3	4	7	2	6	22
	72	2	3	7	1	2	16
	52	2	4	5	2	0	13
	55	2	0	0	0	0	3
	95	1	1	0	1	0	3

59	0	0	1	0	0	1
36	1	0	1	0	0	2
46	1	1	1	0	0	3
63	0	1	0	0	0	1
62	2	0	1	2	1	7
62	1	0	2	1	2	8
43	2	1	0	3	0	7
36	1	1	0	0	0	2
43	2	0	0	3	1	6
48	2	0	0	0	0	3
42	2	1	2	1	0	6
36	0	1	4	1	2	9
64	1	1	1	1	0	4
46	0	0	3	0	0	3
40	0	1	0	0	0	1
38	1	3	0	1	0	6
46	2	2	3	1	0	8
49	1	1	1	1	0	4
46	1	1	2	0	1	6
51	2	1	0	3	0	7
69	2	0	0	0	0	3
35	1	1	1	2	0	6
71	1	1	1	0	0	3
49	1	1	2	1	0	5
66	1	0	1	1	0	3
56	1	0	1	1	0	3
49	1	2	2	1	2	9
89	0	0	3	0	0	4
40	4	1	0	2	0	7
57	0	0	3	0	0	3
58	0	0	0	1	0	1
71	0	0	3	0	0	3
53	0	1	2	1	0	4
55	0	0	1	0	0	1
97	0	0	1	0	0	1
